# Inhibitory effects of *Ficus carica* and *Olea europaea* on pro-inflammatory cytokines: A review

**DOI:** 10.22038/IJBMS.2022.60954.13494

**Published:** 2022-03

**Authors:** Lotfollah Rezagholizadeh, Maryam Aghamohammadian, Mahya Oloumi, Shokofeh Banaei, Mohammad Mazani, Masoud Ojarudi

**Affiliations:** 1 Department of Biochemistry, School of Medicine, Ardabil University of Medical Sciences, Ardabil, Iran; 2 School of Medicine, Ardabil University of Medical Sciences, Ardabil, Iran; 3 Department of Physiology, School of Medicine, Ardabil University of Medical Sciences, Ardabil, Iran; 4 Department of Biochemistry, Faculty of Sciences, Payame Noor University, Tehran, Iran

**Keywords:** Cytokine, Ficus carica, Inflammation, Olea europaea, TNF-α

## Abstract

**Objective(s):**

*Ficus carica *(fig) and *Olea europaea *(olive) are valuable nutritional plants that are widely used in diet and traditional medicine. Different parts of the plants such as fruit and leaves contain beneficial compounds with diverse pharmacological properties, among which anti-inflammatory activities are remarkable. The purpose of this review is to discuss the anti-inflammatory effects of *F. carica* and *O. europaea* with emphasis on their impact on pivotal pro-inflammatory cytokines including IL-1, IL-6, and TNF-α.

**Materials and Methods:**

To prepare the present review, the sites utilized included Scopus, PubMed, Science Direct, and Google Scholar and studied relevant articles from 2000 until 2021.

**Results:**

As a result, we observed that most of the compounds in fig and olive including polyphenols, flavonoids, etc., exert their anti-inflammatory effects through inhibiting or decreasing pro-inflammatory cytokines. Moreover, some natural antioxidants are common between these two plants.

**Conclusion:**

We suggest that consuming figs and olives simultaneously or alone can be useful in the prevention or treatment of inflammatory diseases.

## Introduction

**Figure 1:**
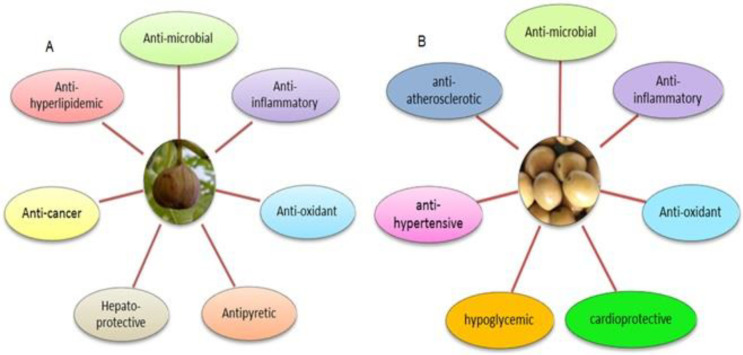
Ficus carica (A) and Olea europaea (B) pharmacological properties

*Ficus carica* (fig) and *Olea europaea* (olive) are beneficial flowering plants that are used in healthy diets and traditional medicine. Many pharmacological properties such as anti-oxidant, anti-inflammatory, anti-cancer, anti-microbial, anti-pyretic, and hepato-protective have been proven about figs and olives (Figure 1), among which this review will focus on anti-inflammatory activities and effects on pro-inflammatory cytokines and mediators (1, 2). 

Inflammation is characterized by the protective response of the immune system against pathogens or endogenous non-infectious molecules. In this process, pro-inflammatory cytokines including Tumor necrosis factor-alpha (TNF-α), Interleukin-1β (IL-1β), interleukin 6 (IL-6), and mediators such as nitric oxide (NO) and prostaglandin E2 (PGE2) increase in body and reactive oxygen species (ROS) are produced (3). Transcription of IL-1, IL-6, and TNF genes are induced by an important protein in the inflammatory process, nuclear factor-kappa β (NF-κβ). Stimulatory agents lead to expression of NF-κβ and then transcription of genes related to pro-inflammatory proteins (4). Several regulatory enzymes involving phospholipase A2 (PLA2), cyclooxygenase (COX-2), and lipoxygenase (LOX) play critical roles in the production of pro-inflammatory mediators. Elimination of foreign/endogenous agents, then, leads to resolution of inflammation and return to hemostasis. However, any failure in this phase leads to prolonged periods of unresolved inflammation and inflammatory disease (3, 5).

Many investigations have been conducted on the anti-inflammatory role of figs and olives and their effects on pro-inflammatory cytokines separately. But studies that include both figs and olives are few. Furthermore, inflammatory disease treatment is a fundamental issue that demands much more importance. According to the World Health Organization (WHO), chronic inflammation and its related diseases are considered a major threat to public health (5). Based on these considerations and according to the significance of consuming natural remedies and preventing unwanted side effects of anti-inflammatory medicines, it is necessary to conduct a study to gather *F. carica* and *O. europaea* anti-inflammatory effects. 

The coronaviruses (CoVs) are single positive-strand RNA viruses that bind to the angiotensin-converting enzyme 2 (ACE2) receptors. The exact mechanism of CoVs infection is not elucidated. The ACE2 receptors are mainly expressed in the alveolar renal epithelial cells, etc. Kidneys, heart, and lungs are highly affected in coronavirus disease 2019 (COVID-19). The viral infection activates the immune response in the host cells that leads to cytokine storm. The cytokine cascade, including interleukins, chemokines, interferons, and TNF-α which causes extensive tissue damage, is correlated with the severity of the infection and associated with the progression of COVID-19 and complications, the important causes of death in COVID-19 patients (6).

The studies indicate that the levels of IL-2, IL-6, IL-7, IL-10, and TNF-α are elevated in COVID-19; moreover, the increased IL-6 levels were found to be associated with disease progression and severe cases. It seems that the cause of mortality in the COVID-19 patients is respiratory failure due to the elevation of cytokine release especially IL-6. The findings report that complications of COVID-19 such as cardio-vascular disorders, acute respiratory distress syndrome (ARDS), renal failure, etc., were associated with the concentration of released cytokines in the blood. A decrease in natural killer T and B cells and increased expression of IL-2, IL6, IL10, TNF-α, and interferon-γ (INF-γ) were observed in the COVID-19 pathophysiology. Also, the increased neutrophil and leukocyte infiltration in COVID-19 patients suggest that targeting the cytokine storm by anti-inflammatory agents may decrease the disease progression and improve the discharge of COVID-19 patients (7). 

Immune reaction to tissue damage and viral infections in the body causes activation of the inflammatory cellular process, which helps the tissue homeostasis under stressed conditions and viral infections such as coronaviruses. Immune response regulates the defense mechanisms against acute pathogens. The cellular mechanism of inflammation includes pro-inflammatory mediators such as cytokines, nuclear factor kappa-B (NF-κβ), TNF-α, interleukins (IL-1, IL-2, IL-6, IL-7, and IL-8), and inflammatory enzymes (cyclooxygenases and lipoxygenase). The inflammatory single transduction is started by the expression of inflammatory mediators, for example, NF-κβ expression in COVID-19 patients. NF-κβ binds to different regions of several genes and plays a major role in various inflammatory diseases. The expression of cyclooxygenase (COX)-2, nitric oxide synthase (NOS), interleukins, and TNF-α is modulated through NF-κβ. Various studies have shown that the oxidative stress induced by expression of NOS and COX results in more activation of inflammatory factors. Also, the activation of a single transducer and activator of transcriptions 3 (STAT3), a transcriptional factor is regulated by Janus activated kinase (JAK) which results in phosphorylation and translocation of STAT3 where it binds to the DNA and causes expression of various inflammatory factors. Therefore, inflammatory blockers such as figs and olives can control acute inflammatory processes and prevent overexpression of the cytokines that have critical roles in the pathophysiology of inflammatory disorders and COVID-19 (8). 

## Methods

The current study has been conducted using searches for scientific data published about phytochemistry, pharmacological activities, and anti-inflammatory compounds of *F. carica* and *O. europaea*. The sites utilized included Scopus, PubMed, Science Direct, and Google Scholar. To this end, the papers published in English and during the years 2000–2021 were studied. In this review, keywords such as *Ficus carica*, *Olea europaea*, inflammation, TNFα, IL-6, IL-1, NF-kB, disease, and treatment are used. 

## Results

***Ficus carica***

**Figure 2:**
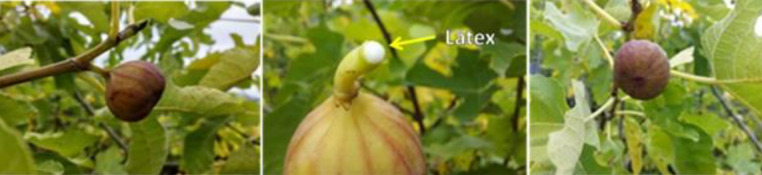
Ficus carica plant (fruit, leaves, and latex)

*F. carica* contains a large amount of minerals, carbohydrates, vitamins, sugars, dietary fiber, organic acids, and phenolic compounds. Fruit, leaves, and latex of *F. carica* have diverse anti-inflammatory contents (Figure 2). Phytochemical analyses show that the fruit contains alkaloids, tannins, glycosides, flavonoids, saponins, coumarins, phenols, sterols, terpenes, carbohydrates, and proteins (9).

The compounds found in leaf extract are phenolic compounds, flavonoids, organic acids, resin, sterol, carbohydrates, and lipids. Also, latex has a large amount of polyphenols, flavonoids, and anthocyanins (10).

Flavonoids are polyphenolic compounds that are found in fruit, leaves, and latex of *F. carica*. They are divided into different subclasses including flavonols, flavanones, flavones, isoflavone, and anthocyanidins. Some types of these subclasses are responsible for anti-inflammatory roles in the fig fruit involving quercetin, luteolin, apigenin, hesperetin, catechin, and cyanidin (11, 12). 

In a recent phytochemical investigation on the fig fruit, 16 prenylated isoflavone derivatives were isolated and confirmed to have remarkable anti-inflammatory effects due to their inhibitory action against NO production(13). 

Flavonoids morin, hesperetin, and rutin were shown to be effective in reducing inflammatory cytokines IL-1β, IL-6, and TNF-α in diabetic animals (5). The flavonoid quercetin was revealed to inhibit bacterial LPS-induced iNOS and TNF-α secretion in macrophages, LPS-induced IL-1β and TNF-α secretion in RAW 2647. Furthermore, quercetin reduced TNF-α and IL-6 in the mice model (5). In a study on human triple-negative cells, apigenin of flavones found in the fig fruit inhibited the activation of immune cells and down-regulated TNF-α mediated up-regulation of IL-1α and IL-6. Apigenin is a relatively small molecule that has been shown to cross the blood-brain barrier and enter the cerebrospinal fluid compartment, so it can be utilized for CNS inflammation treatment (5).

Anthocyanidins exert anti-inflammatory activity by inhibition of COX-2 expression in LPS-activated RAW 264 cells or inhibiting inducible iNOS protein and mRNA expression in LPS-activated murine J774 macrophages (14). Cyanidin-3-o-β-D-glucoside, of anthocyanins found in the fig fruit, inhibits inflammatory pathways through ROS inhibition and suppression of NF-κβ and its dependent genes in animal models (5).

Sharma *et al*. explored that morin, a flavonol found in the fig fruit, significantly decreased the expression of TNF-α, IL-6, COX-2, and PGE2 in 1,2-dimethylhydrazine (DMH)-induced colon cancer by down-regulating NF-κβ pathway, thus acting as a potent anti-inflammatory agent (15). 

*F. carica* leaf extract contains high total phenolic content that inhibits the production of pro-inflammatory cytokines including TNF-α and PGE2 (16). Gene expression of TNF-α and IL-1α, being analyzed in human keratinocyte cells (HaCaT) using RT-qPCR by Turkoglu *et al*. showed that plant extract of *F. carica* caused statistically significant down-regulation of these factors due to its phenolic compounds (17). 

Lupeol is a naturally occurring triterpene that exists in *F. carica* leaves and has immunomodulating properties (18). In a study carried out on LPS-treated macrophages, lupeol was reported to decrease the generation of pro-inflammatory cytokines such as TNF-α and IL-1β (19). Moreover, leaves are characterized by higher quantities of psoralen and bergapten, furocoumarin subclasses (20, 21). Bergapten plays its anti-inflammatory role by decreasing LPS-induced production of pro-inflammatory cytokines and mediators, inhibiting LPS-elevated expressions of iNOS and COX-2, and suppressing LPS-stimulated ROS production and activation of the JAK-STAT signaling pathway (22). A recent study revealed the potential benefit of bergapten in managing allergy-related inflammatory conditions (23). 

*F. carica* latex is a natural source of psoralen. In a study on periodontitis, psoralen could attenuate the inflammatory response and inhibited LPS-induced IL-1β and IL-8 mRNA expression (20, 24). Ficin, a unique enzyme derived from fig latex was investigated by researchers and proved to inhibit NO and iNOS protein expression and suppress phosphorylation of Iκβ/NF-κβ in LPS-stimulated RAW264 cell. Ficin can inhibit phosphorylation of MAPK and STAT3 protein which are linked to IL-6 receptor and exert anti-inflammatory activities (25). Table 1 presents *F. carica* compounds with their anti-inflammatory effects.

***Olea europaea***

**Figure 3:**
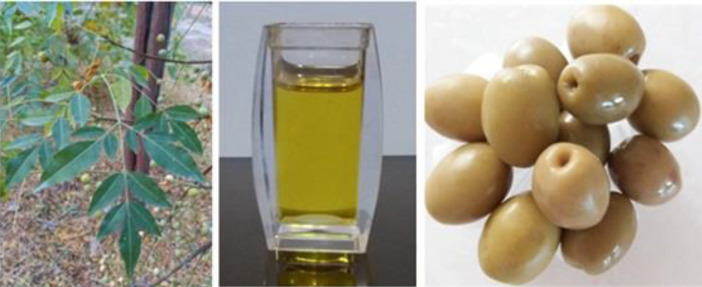
*Olea europaea* (olive leaves, oil, and fruit)

Olive fruit is composed of many beneficial compounds including water, protein, oil, carbohydrates, cellulose, inorganic substances, pigments, pectin, organic acids, and phenolic compounds (Figure 3). Components that were reported to possess anti-inflammatory effects in olive fruit are oleuropein, hydroxytyrosol, luteolin, rutin, apigenin, verbascoside, quercetin, coumaric acid, caffeic acid, anthocyanins, hesperidin, etc. (26, 27).

Oleuropein, a glycosylated secoiridoid, is a type of phenolic bitter compound found in olive fruit and leaves, and one of the main components responsible for anti-inflammatory effects in *O. europaea* (28, 29). Oleuropein has the ability to decrease inflammatory cytokines (IL-6, TNF-α, and IL-1β) and reduce NF-κβ activation (30, 31). Modulating the MAPK signaling pathway, scavenging superoxide anions, and inhibiting hypochlorous acid-derived radicals are other activities that have been reported about oleuropein (32, 33). It inhibits several inflammatory enzymes such as lipoxygenases and prevents free radical formation by chelating metal ions which catalyze free radical production reactions (38). Based on reports, only oleuropein has a significant inhibitory effect on TNF-α production at low concentrations (40).

Hydroxytyrosol, a small phenolic molecule found in aqueous olive fruit and leaf extract, is the principal degradation product of oleuropein (32, 34). Hydroxytyrosol reduces the secretion of IL-1α, IL-1β, IL-6, IL-12, and TNF-α and expression of iNOS, PGE2 synthase (PGES), and COX-2 (30, 35). Hydroxytyrosol has been reported to suppress the translocation of NF-κβ to the nucleus in the LPS-induced human monocytic THP-1 cells (34), thus it suppresses the NF-κβ signaling pathway. It also scavenges free radicals and reduces NO and PGE2 production (35). Furthermore, *in vivo* studies indicate that hydroxytyrosol exerts anti-inflammatory effects through modulating MAPK signaling (33). 

Coumaric acid is a natural phenolic acid that exists in olive fruit, oil, and leaves (26). Several studies have revealed the anti-inflammatory properties of coumaric acid including a recent study carried out on cigarette smoke extract-activated epithelial cells, coumaric acid decreased the production of IL-8 as efficiently as dexamethasone (36). The work of Kheiry *et al*. showed that the elevated levels of TNF-α and IL-6, and ROS production in LPS-induced lung inflammation were decreased by coumaric acid in rats (37). However another study suggested that phenolic acids without catechol groups such as coumaric acid, do not have anti-inflammatory effects, but compounds containing catechol groups such as chlorogenic acid and caffeic acid exert anti-inflammatory effects (38-40). They showed that chlorogenic acid and caffeic acid, containing the catechol group, inhibit IL-8 production (41). 

Virgin olive oil, which is extracted from the fruit, is rich in high-value compounds such as phenolics, phytosterols, tocopherols, triterpene dialcohols, squalene, carotenoids, and chlorophyll (26). In recent research on olive oil polyphenols, oxysterols-induced pro-inflammatory responses in intestinal cells were inhibited by polyphenols through modulating NF-κβ activation, inhibiting iNOS induction and NO release. Olive oil phenolic extract inhibits IL-8, IL-6 production, and oxidant species formation (33). Extra virgin olive oil has the ability to decrease the levels of IL-1β, IL-6, IL-8, and TNF-α through down-regulation of the NF-κβ pathway (30, 42). 

Oleanolic acid, erythrodiol, and uvaol are pentacyclic triterpenes, found in olive pomace oil which exert their anti-inflammatory effects by decreasing IL-1β and IL-6 production in a dose dependent manner. In an investigation, all three compounds were demonstrated to reduce TNF-α production significantly (43).

Secoroidoids including oleacein and oleocanthal are the most abundant family of phenolic compounds in extra virgin olive oil. Oleacein decreases inflammation by reducing COX-2 levels and attenuating the NF-κβ signaling pathway. Also, oleacein has been reported to inhibit arachidonate 5-lipoxygenase, which is responsible for the pro-inflammatory leukotriene biosynthesis. The other compound, oleocanthal, has anti-inflammatory and anti-oxidant properties similar to the non-steroidal drug ibuprofen. It has the ability to inhibit COX-1 and COX-2. Furthermore, it can inhibit the expression and production of IL-6, IL-1β, TNF-α, and iNOS based on results obtained from several studies (44, 45). 

Olive leaf extract has a higher concentration of polyphenol compounds compared with extra virgin olive oil. Iridoids (a type of monoterpenoids) and flavonoid contents act as the main anti-inflammatory agents in olive leaves (31, 46). According to studies, olive leaf extract decreases IL-6, IL-8, IL-1β, and TNF-α levels. In a study carried out on peripheral blood mononuclear cells stimulated with LPS, IL-8 was expressed less and serum IL-8 levels decreased after consuming OLE for 6 weeks. OLE also inhibits COX-2 expression and down-regulates NF-κβ and arachidonic acid pathways involved in the inflammation process (31, 47, 48). Table 2 presents *O. europaea* compounds and their anti-inflammatory effects.

By comparing the composition of *F. carica* and *O. europaea*, we observed some compounds including apigenin, luteolin, quercetin, catechin, rutin, hesperidin (aglycone form: hesperetin), anthocyanin, and tocopherol, all of which have anti-inflammatory effects common in both of them. Thus, consuming the combination of *F. carica* and *O. europaea* may have remarkable results in reducing inflammatory cytokines and mediators during treatment of inflammatory diseases such as asthma, tuberculosis, rheumatoid arthritis, periodontitis, ulcerative colitis, sinusitis, atopic dermatitis, allergies, etc. 

**Table 1:** *Ficus carica compounds *with their anti-inflammatory effects

Compounds	Part of plant	Effects	References
Apigenin	Fruit	Decreasing ROS, COX-2, iNOS, PGE2; reducing IL-1β, IL-6, TNF-α, and IL-8; inhibiting NF-κβ and AP-1 transcription factors.	[5,51]
Luteolin	Fruit	Inhibiting IL-1β, IL-8, IL-6, and TNF-α; restraining NF-κβ pathway, iNOS expression, and NO production; scavenging ROS.	[5,52]
Quercetin	Fruit	Inhibiting production of IL-1β, IL-6, and TNF-α; decreasing COX-2 and iNOS.	[5,53]
Hesperetin	Fruit	Decreasing IL-1β, TNF-α, NF-κβ, COX-2, iNOS, and ROS.	[5]
Prenylated Isoflavones	Fruit	Inhibitory effects against NO production	[13]
Morin	Fruit	Suppressing the NF-κβ pathway; reducing the expression of pro-inflammatory cytokines (IL-6, TNFα), COX-2, and PGE2.	[15,54]
Anthocyanins (Cyanidin)	Fruit	Inhibiting COX-2 and NF-κβ expression, scavenging free radicals.	[11,14]
γ-tocopherols	Fruit	Reducing inflammation	[16]
Phenolic compounds	Leaves, Latex	Inhibiting production of pro-Inflammatory cytokines; decreasing ROS production by phagocytic cells; decreasing MPO activity	[16,55]
Steroidal sapogenins	Leaves	Reducing IL-1β, IL-6, and NO production.	[55,56]
Ficusogenin	Leaves	Anti-inflammatory	[11]
Bergapten	Leaves	Decreasing pro-inflammatory cytokines (IL- 1β, TNF-α, and IL-6); inhibiting expression of iNOS and COX-2; suppressing ROS production and JAK-STAT signaling pathway.	[22,20]
Lupeol	Leaves	Decreasing generation of pro-inflammatory cytokines (TNF-α, IL-1β)	[11,19]
Psoralen	Leaves, Latex	Inhibiting IL- 1β and IL-8 mRNA expression.	[20,24]
Phthalic acid	Latex	Anti-inflammatory effects	[20]
Catechin	Fruit, Latex	Reducing inflammatory mediators and cytokines (TNF-α, IL-1β); decreasing expression of iNOS and COX-2, Inhibiting NO production; reducing NF-κβ.	[5,57]
Rutin	Latex	Suppressing expression and release of IL-6, TNF-α, IL-1β, COX-2, iNOS, and NF-κβ.	[58-60]
Ficin	Latex	Inhibiting NO and iNOS expression; suppressing phosphorylation of Iκβ/NF-κβ, MAPK, and STAT3	[25]

AP-1: Activator protein 1; COX-2: Cyclooxygenase 2; IL-1β: Interleukin 1 beta; IL-6: Interleukin 6; IL-8: Interleukin 8; iNOS: inducible nitric oxide synthase; JAK_STAT: Janus kinase signal transducer and activator of transcription; MPO: Myeloperoxidase; NF-κβ: Nuclear factor-kappa β; NO: Nitric oxide; PGE2: Prostaglandin E 2; ROS: Reactive oxygen species; STAT3: Signal transducer and activator of transcription 3; TNF-α: Tumor necrosis factor-alpha

**Table 2:** Olea europaea compounds with their anti-inflammatory effects

Compounds	Part of plant	Effects	References
**Apigenin**	Fruit, leaves, oil	Decreasing ROS, COX-2, iNOS, MAPK; reducing IL-1β, IL-8, IL-6, TNF-α, PGE2.	[5,26]
**Luteolin**	Fruit, leaves, oil	Inhibiting IL-1β, IL-8, IL-6, TNF-α; restraining NF-κβ pathway, iNOS expression, and NO production; scavenging ROS; decreasing MAPK and COX-2.	[5,52,26]
**Quercetin**	Fruit, leaves	Inhibiting production of IL-1β, IL-6, and TNFα; inactivating NF-κβ; down-regulating phosphorylation of MAPK; suppressing expression of COX-2 and iNOS.	[5,53,57,32]
**Oleuropein **	Fruit, oil, leaves	Decreasing IL-1β, IL-6, TNF-α; reducing NF-κβ activation; scavenging superoxide anions; modulating MAPK signaling pathway; inhibiting lipoxygenases.	[30-33,48]
**Hydroxytyrosol**	Fruit, oil, leaves	Decreasing secretion of IL-6, IL-1α, IL-1β, IL-12, and TNF-α, and expression of iNOS, PGE2 synthase, and COX-2; modulating MAPK signaling pathway and suppressing NF-κβ pathway; decreasing production of PGE2 and NO.	[30,34,35]
**Verbascoside **	Fruit, leaves	Inhibiting IL-6, IL-8, IL-12 and TNF-α by suppressing NF-κβ pathway; inhibiting ROS and NO production.	[61-64]
**Coumaric acid**	Fruit, oil, leaves	Suppressing MAPK and NF-κβ pathways; decreasing IL-6, IL-8, TNF-α and IL-1β; decreasing ROS production inhibiting iNOS and COX-2.	[37,65,36]
**Diosmetin**	Leaves	Inhibiting TNF-α production, suppressing NF-κβ pathway	[66,67]
** Uvaol**	Oil, leaves	Decreasing mRNA expression of IL-1β, IL-6, TNF-α, iNOS, and COX-2; inhibiting NO production	[68,69]
**Oleanolic acid**	Oil, leaves	Decreasing IL-1β, IL-6 and TNF-α; suppressing COX-2 expression; regulating NF-κβ signaling pathway	[70,71,43]
**erythrodiol**	Oil, leaves	Decreasing IL-1β, IL-6, TNF-α; suppressing ROS	[72-74]
**Maslinic acid**	Oil, leaves, fruit	Decreasing IL-1β, IL-6 , TNF-α, and NO	[67,75,76]
**Kaempferol **	Leaves, oil	Inhibiting COX-2 and iNOS expression; suppressing the NF-κβ pathway	[32,77-79]
**Gallic acid**	Fruit, leaves, oil	Decreasing TNF-α by suppressing the NF-κβ pathway; regulating NOX1 expression; decreasing NOX2 expression	[80,48,81,82]
**Caffeic acid**	Leaves, fruit	Suppressing activation of NF-κβ signaling; decreasing ROS production and PGE2 expression; reducing IL-6, IL-8 and TNF-α production.	[83,41]
**Chlorogenic acid **	Leaves, fruit	Suppressing NF-κβ transcription; scavenging ROS; inhibiting IL-8 production.	[41]
**Catechin**	Leaves	Suppressing expression of iNOS, COX-2, TNF-α, and IL-1β; inactivating NF-κβ; down-regulating phosphorylation of MAPK.	[32,57]
**Rutin**	Leaves	Suppressing expression and release of IL-6, TNF-α, IL-1β, COX-2, iNOS, and NF-κβ.	[32,58-60]
**Oleocanthal**	Oil	Reducing expression of IL-6 mRNA and secretion of IL-1β, IL-6, and TNF-α; inhibiting COX-1 and COX-2.	[30,84]
**Cinnamic acid**	Leaves	Suppressing the NF-κβ pathway.	[85,86]
**Ellagic acid**	Leaves	Inhibition of TNF-α and IL-1β production and suppressing pro-inflammatory intervention with NF-κβ pathway; decreasing COX-2 expression.	[87-89]
**Anthocyanin**	Fruit, leaves, oil	Inhibiting IL-1β, IL-6, TNF-α, and expression of COX-2 and iNOS; decreasing ROS level.	[90,91]
**Hesperidin **	Leaves	Decreasing IL-1β, IL-6, TNF-α; inhibiting NO.	[92,5]
**α-tocopherol**	Oil, leaves	Inhibiting production of IL-6, IL-1β, and TNF-α; blocking the expression of COX-2 and iNOS.	[93,94]
**Oleacein **	Leaves, oil	Attenuating NF-κβ pathway; inhibiting TNF-α and ROS expression; decreasing COX-2 level; inhibiting NO, iNOS, and NOS2 production.	[45,95-97]

**Figure 4:**
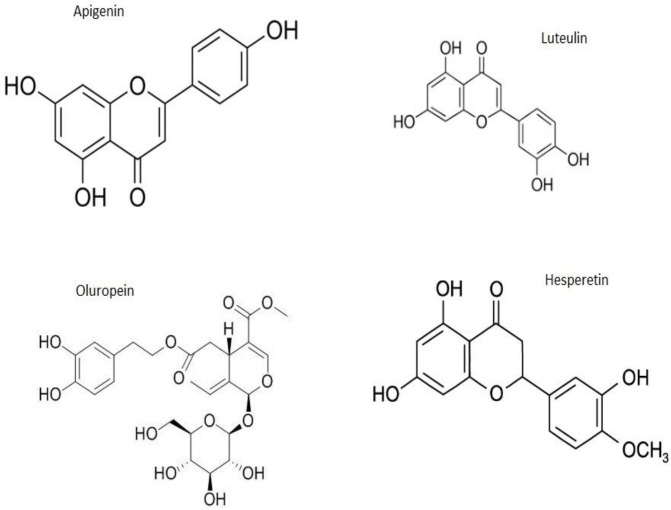
Chemical structures of apigenin, luteulin, oleuropein, and hesperetin

## Discussion

Here, we reviewed anti-inflammatory effects of *F. carica* and *O. europaea* along with their natural components on pro-inflammatory cytokine expression. Figure 4 shows the chemical formula of several important compounds of figs and olives. Previous research suggests that these medicinal plants have anti-oxidant and anti-inflammatory properties, consistent with these findings, we compared the results of studies on the role of figs and olives in decreasing or inhibition of pro-inflammatory cytokines. The compounds of fruit including apigenin, luteolin, quercetin, and hesperetin result in reduction of ROS and RNS production, scavenging, and decrease of interleukin expression. Therefore, the compounds can be considered in the treatment of many types of inflammatory diseases and syndromes (32). 

Coronavirus disease 2019 (COVID-19) caused by coronavirus-2 leads to acute respiratory syndrome; the virus infects the respiratory epithelial cells and other systems via angiotensin-converting enzymes2 (ACE2) receptors. Recent findings demonstrate that a cytokine cascade results in severe inflammation and thrombotic events. Therefore, covid-19 patients experiencing immunological diseases and inflammatory conditions receive immunomodulatory and anti-inflammatory drugs and an anti-inflammatory diet. There are various data about the roles of inflammation and thrombosis in the disease prognosis. Also, inflammatory pathways involved in the pathogenesis of covid-19 reveal lymphocyte infiltration and cytokine releasing syndromes (49). 

COVID-19 respiratory distress begins with an early inflammation so that pulmonary compliance is reduced by activation of intercellular inflammatory pathways, cytokine synthesis, or storm (excessive release of IL-2, IL-6, IL-7, IL-8, IL10, and TNF-α). Furthermore, activated T lymphocytes and natural killer cells result in increased release of interleukins, interferon-γ (INF-γ), and activation of the apoptotic pathways. Finally, this vicious cycle leads to pulmonary edema and respiratory distress syndrome, and triggered inflammation promotes the coagulation system leading to changes in coagulation factors and thrombosis. A systemic inflammatory syndrome induced by the release of pro-inflammatory cytokines accelerates cellular damage in different tissues such as the lungs, kidneys, brain, and other tissues in patients with COVID-19 (50). 

As mentioned, anti-inflammatory drugs, decreasing inflammatory diet, and immunomodulatory therapy are critical therapeutic interventions in the pathophysiology of inflammatory diseases and COVID-19. Thus, this review summarized anti-inflammatory mechanisms and properties of figs, olives, the compounds found in fruit, leaves, and latex along with their potential therapeutic effects in the management of beneficial treatment in the inflammatory conditions and diseases such as tuberculosis, rheumatoid arthritis, inflammatory bowel diseases (IBD), sinusitis, allergies, etc.

We suggest that consumption of figs and olives may be useful in the improvement of inflammatory symptoms and clinical outcomes. Moreover, ellagic acid, caffeic acid, quercetin, tocopherols, flavonoids, etc., found in figs and olives, which are known anti-inflammatory and anti-oxidant agents, are suggested for further studies in order to clarify the exact mechanisms mediating the efficacy of fig and olive combination therapy in the inflammatory diseases and COVID-19.

## Conclusion

This review presented *F. carica* and *O. europaea* (fruit, leaves, and latex) effects on pro-inflammatory cytokines. *F. carica* and *O. europaea* are rich sources of compounds with anti-inflammatory activities and can be good choices for prevention/treatment of inflammatory disease. Furthermore, *F. carica* and *O. europaea* contain some of these compounds in common. In light of these explanations, we suggest that a combination of *F. carica *and* O. europaea* may have synergistic effects in suppressing inflammatory cytokines and mediators. However, *in vivo* clinical studies are required to support these findings and reveal more results related to this study.

## Authors’ Contributions

MA and MO Wrote the manuscript; LR Designed the study; SB Designed the study and wrote the manuscript. All authors read and approved the final manuscript.

## Conflicts of Interest

None.
